# Analysis of Femtosecond Laser Assisted Capsulotomy Cutting Edges and Manual Capsulorhexis Using Environmental Scanning Electron Microscopy

**DOI:** 10.1155/2014/520713

**Published:** 2014-11-20

**Authors:** Sebastiano Serrao, Giuseppe Lombardo, Giovanni Desiderio, Lucio Buratto, Domenico Schiano-Lomoriello, Marco Pileri, Marco Lombardo

**Affiliations:** ^1^Fondazione G.B. Bietti IRCCS, 00198 Rome, Italy; ^2^CNR-IPCF Unit of Support of Cosenza, 87036 Rende, Italy; ^3^Vision Engineering Italy Srl, 00198 Rome, Italy; ^4^CAMO SpA, 20121 Milan, Italy; ^5^Azienda Ospedaliera San Giovanni-Addolorata, 00100 Rome, Italy

## Abstract

*Purpose*. To investigate the structure and irregularity of the capsulotomy cutting edges created by two femtosecond (FS) laser platforms in comparison with manual continuous circular capsulorhexis (CCC) using environmental scanning electron microscopy (eSEM). *Methods*. Ten anterior capsulotomies were obtained using two different FS laser cataract platforms (LenSx, *n* = 5, and Victus, *n* = 5). In addition, five manual CCC (*n* = 5) were obtained using a rhexis forceps. The specimens were imaged by eSEM (FEI Quanta 400, OR, USA). Objective metrics, which included the arithmetic mean deviation of the surface (Sa) and the root-mean-square deviation of the surface (Sq), were used to evaluate the irregularity of both the FS laser capsulotomies and the manual CCC cutting edges. *Results*. Several microirregularities were shown across the FS laser capsulotomy cutting edges. The edges of manually torn capsules were shown, by comparison of Sa and Sq values, to be smoother (*P* < 0.05) than the FS laser capsulotomy edges. *Conclusions*. Work is needed to understand whether the FS laser capsulotomy edge microirregularities, not seen in manual CCC, may act as focal points for the concentration of stress that would increase the risk of capsular tear during phacoemulsification as recently reported in the literature.

## 1. Introduction

Several femtosecond (FS) laser platforms have been developed to automate certain steps during cataract surgery, such as clear corneal incision (CCI), capsulotomy, and nucleus fragmentation [1–5]. An increasing number of studies are reporting data on the safety and efficacy of FS laser platforms to create anterior capsulotomy [6–8]. Overall, the FS laser capsulotomies have been shown to be better centered than manual CCC, with highly predictable diameters [1, 7, 9]. Previous studies suggest that optimal capsulotomy centration and accurate size may maximize the performance of premium intraocular lenses (IOLs) [10, 11]. Nevertheless, recent work [12] has reported greater incidence of capsular tears using FS commercial laser platform, compared to conventional phacoemulsification surgery, even beyond the initial learning curve expected with the technology [6]. Several studies [8, 12–15] have analyzed the lens capsulotomies using either optical or scanning probe microscopy techniques. The light optical microscopy has been mainly used to monitor lens epithelial cell (LEC) death after capsulotomy. High-resolution scanning electron microscopy (SEM) imaging has revealed irregularities at the capsulotomy edges, which have not been observed in manual capsulorhexis (CCC). Overall, variations in laser settings have been shown to affect the morphology of capsulotomy cutting edges [12–15]. It is therefore of great interest to understand the source(s) of these microstructural irregularities and how to prevent them to optimize the surgical outcome of FS laser assisted cataract surgery.

The purpose of our study was to analyze, using environmental scanning electron microscopy (eSEM), the morphology and irregularity of the capsulotomy cutting edges obtained using two FS commercial laser cataract platforms, in comparison with manual CCC.

## 2. Materials and Methods

Fifteen consecutive patients (mean age: 65 ± 5 years) diagnosed with senile corticonuclear cataract and no other concurrent eye pathologies, including no corneal opacity, were recruited. The study followed the tenets of the Declaration of Helsinki and all patients signed an informed consent after full explanation of the procedure.

The femtosecond laser assisted cataract surgeries were performed by an experienced surgeon (Lucio Buratto) using two FS laser cataract platforms, which included the LenSx (Alcon Laboratories Inc., Fort Worth, TX, USA; *n* = 5) and the Victus (Bausch & Lomb Inc., Dornach, Germany; *n* = 5). The intended capsulotomy diameter was 5.50 mm in all cases. Integrated real time OCT was used to plan and monitor the capsulotomy cut using both FS laser platforms.

The LenSx laser has a repetition rate of 33 kHz, pulse width of 600 to 800 fs, and central laser wavelength of 1030 nm. The proprietary rigid curved interface was used in all cases. The laser energy was set to 15 *μ*J with spot and layer separations of 3 and 4 *μ*m, respectively. The Victus laser has a pulse frequency up to 80 kHZ, pulse duration of 400 to 500 fs, and central wavelength of 1040 nm. The curved patient interface provided by the manufacturer was used in all cases. The laser energy setting was 7.0 *μ*J with spot and layer separations of 6 and 4 *μ*m, respectively.

The conventional phacoemulsification was performed by one experienced surgeon (Sebastiano Serrao). Manual capsulorhexis was created using a rhexis forceps (*n* = 5).

The fifteen anterior lens capsules, collected during cataract surgery, were immediately fixed in 2.5% glutaraldehyde solution and stored at 4°C for 24 hours. The specimens were then scanned by eSEM (FEI Quanta 400, OR, USA) without any further sample processing. Imaging was performed using 5.9 mbar vacuum and 3°C temperature in the eSEM chamber, after fixing each sample to the microscope holder with a drop of silver nitrate. The images were acquired at 10 kV voltage (HV), using magnifications (Mag) ranging from 400x to 6000x. The technician (Giovanni Desiderio) was masked as to which type of capsulorhexis was performed.

Image analysis was performed using ImageJ (http://imagej.nih.gov/ij/, NIH, USA). The irregularity measurements were performed on several reference areas of 7 × 7 *μ*m along the capsular cutting edge in images acquired at 3000x Mag. The areas studied have been randomly chosen (at least 4 areas for capsule). Four quantitative parameters were used to characterize the cutting edge irregularity, which included the arithmetic mean deviation of the surface (Sa), the root-mean-square deviation of the surface (Sq), the skewness of the topography height distribution (Ssk), and the kurtosis of the topography height distribution (Sku). Detailed description of the above parameters has been given in previous work [16]. The surface skewness (Ssk) describes the asymmetry of the surface height distribution. Negative Ssk (Ssk < 0) indicates a predominance of valleys and positive Ssk (Ssk > 0) a predominance of peaks. If Ssk = 0, a symmetric height distribution is indicated (i.e., Gaussian like). In general, if the Ssk is >1 or <−1, the skewness is substantial and the distribution is far from symmetrical. The surface kurtosis (Sku) quantifies whether the shape of the data distribution matches the Gaussian distribution. Overall, kurtosis represents a measure of the randomness of surface heights. For normal height distributions, Sku = 3; for spiky distributions, Sku > 3; for bumpy distributions, Sku < 3.

## 3. Statistics

The one-way analysis of variance (ANOVA) test was used to statistically compare the differences among the anterior lens capsular edges characteristics. When statistical significance was found, the differences between the cutting edges were further compared using the Tukey test for pairwise comparisons. Differences with a *P* value of 0.05 or less were considered statistically significant.

## 4. Results

The surgery was uneventful in all cases. No tears or incomplete procedures were recorded. Five anterior lens capsules were created by the LenSx laser, five samples were obtained using the Victus laser, and five samples were obtained by manual CCC.

**Figure 1:**
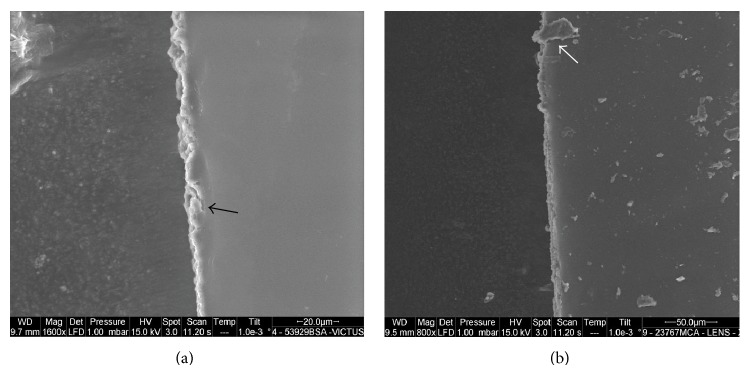
(a), (b) The Victus and LenSx capsulotomy cutting edges are shown, respectively. Linear cracks (black arrow) and tags (white arrow) are spread across the edges. The generation of these features may be consistent with eye movements during laser capsulotomy.

**Figure 2:**
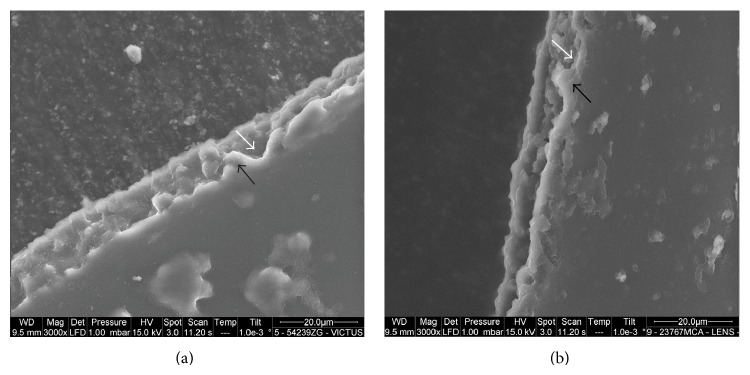
(a), (b) The Victus and LenSx capsulotomy cutting edges are shown, respectively. The microirregularities at the capsular edge are a direct consequence of the FS laser photodisruptive effects. Bumps (black arrows) and notches (white arrows) were spread across the cutting edges, giving rise to a variable pattern, ranging from a can opener to a postage stamp-like structure, that are consistent with cavitation and thermal effects of FS laser on the capsule microstructures.

**Figure 3:**
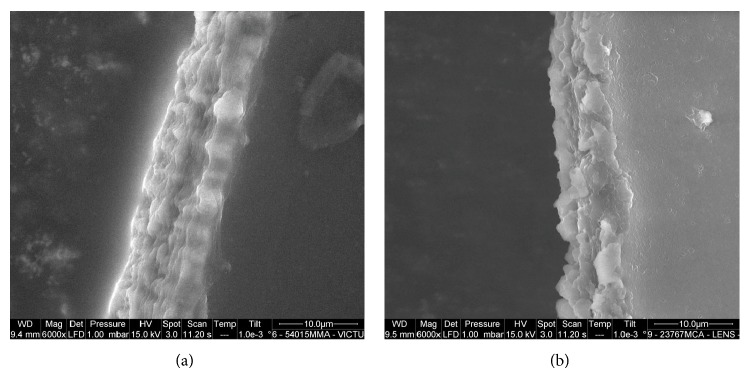
(a), (b) High-magnification images of the Victus and LenSx capsulotomy cutting edges, respectively. The stacks of collagen fibers at the capsular edge could be seen in some specimens (3/5) treated by the Victus FS laser. This was not the case for the LenSx specimens, likely related to a high thermal effect excerpted on the collagen fibers.

**Figure 4:**
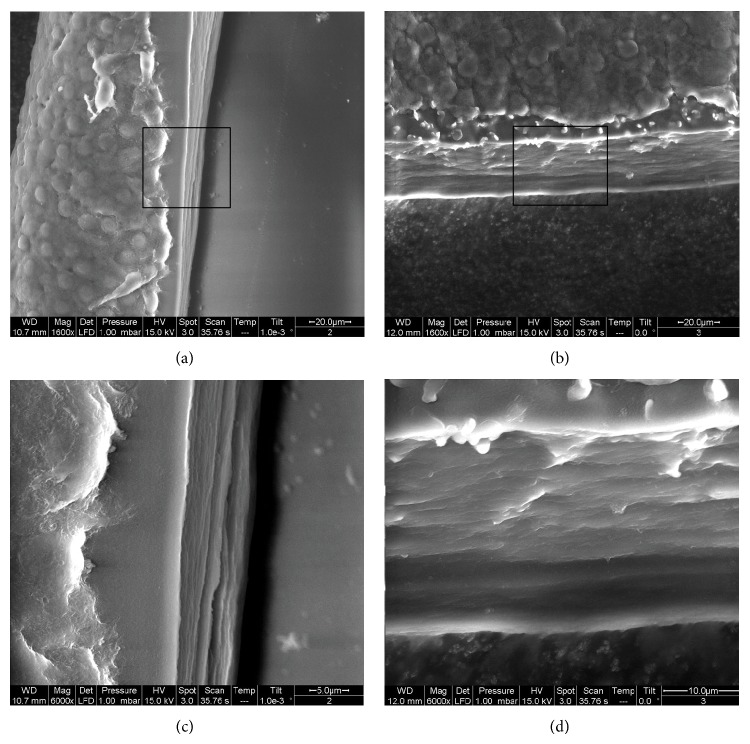
(a), (b) Two manually torn capsules are shown. The stacks of collagen fibers are clearly shown at the edge of the capsule. The edge morphology is smooth, with no irregularity. (c), (d) High-magnification images of the manual CCC edges are shown in (a) and (b), respectively.

The LenSx capsulotomy cutting edges showed a postage stamp like pattern, with several bumps and notches of variable width, ranging between 3 and 10 *μ*m, that were spread across the edge (Figures 1, 2, and 3). Linear cracks (length ranging between 4 and 9 *μ*m; width < 3 *μ*m) were also seen across the edges. The Victus capsulotomy cutting edges showed micro-can opener structure; nevertheless the stacks of collagen fibers could be seen at the capsular edges in some specimens (Figures 1, 2, and 3). Linear cracks (width < 3 *μ*m) and notches (width ranging between 3 and 9 *μ*m) were also seen at the capsular edge. The manual CCC cutting edges showed clear stacks of collagen fibers (Figure 4). The edges were smooth and regular in all cases. No microdiscontinuities of the edge were seen in any case.

The manual CCC had statistically significantly smoother cutting edges than FS laser capsulotomies, as summarized in Table 1. Both the Sa and the Sq values were statistically significantly lower (*P* < 0.05) in manual CCC than in FS capsulotomy cutting edges. There were no differences in Sa and Sq values (*P* > 0.05) between the two FS laser platforms. The distribution of data showed moderate skewness, with average values ranging between 0.35 and 0.62 across specimens. No significant differences in Ssk values (*P* > 0.05) were found between the FS laser capsulotomies and the manual CCC cutting edges, however, with the laser-cut capsules showing high intersample variation. The Sku values were less positive (Victus) or showed negative values (LenSx), in the FS laser samples, indicating a flatter height distribution than manual CCC.

In manual CCC, the LEC boundary was close to the cutting edge, with mean distance of 12 ± 8 *μ*m. It was on average 40 ± 9 *μ*m far from the laser capsulotomy cutting edge, with no differences between the FS laser platforms.

## 5. Discussion

In conventional phacoemulsification, the CCC is performed manually by using a rhexis forceps and generating shearing and tearing forces centripetally, thus counterbalancing the centrifugal forces from the zonules [17, 18]. In general, the capsule flap is regrasped every 3 to 4 clock hours to further minimize the contribution of centrifugal forces. It is challenging to repeatedly achieve a well-centered CCC of 5/5.5 mm even by the most experienced cataract surgeon. FS cataract laser platforms are showing increased predictability to obtain capsulotomies that are round, well centered, and of the desired size in comparison with manual CCC [6–9]. After docking the patient interface, the capsulotomy size and position are set according to the pupillary aperture using real-time high-resolution anterior segment imaging (either OCT or Scheimpflug imaging). Focal photodisruption of the anterior lens capsule generates multiple overlapping craters at the capsulotomy edge. At the end of the FS laser assisted procedure, the capsulotomy leaf is gently pulled centripetally with a forceps.

The human lens capsule is considered a specialized basement membrane. The major structural component of the lens capsule is basement membrane type IV collagen, which is organized into a three-dimensional molecular meshwork [19–22]. Accordingly, the human lens capsule has intrinsic elasticity [23–25]. The tensional force that tears off the capsule during manual CCC is directed in the same direction tangentially to the edge. On the contrary, the FS laser pulses cut perpendicularly the anterior capsule. The force that induces the manual capsulorhexis is oriented in one unique direction while the laser capsulotomy is the resultant of a sequence of circular oriented multiple spots [26]. Photodisruption effects at the capsulotomy edge disrupt the normal collagen arrangement of the anterior lens capsule and induce irregularities, as shown in the present and previous work [12–15]. The microirregularities at the capsulotomy edge have been found in all the commercial FS laser platforms. Previous work [12] showed the structure of laser-cut capsules obtained using three FS laser platforms, which included the LenSx, the Lensar, and the Catalys. Here we also showed the morphology of capsulotomy edges obtained using the Victus.

In this study, we compared the microstructure and irregularity of the FS laser capsulotomy edges in comparison with manually torn capsular edges. The eSEM images have not the surface artifacts occuring during sample preparation in conventional SEM imaging, such as graded alcoholic dehydration followed by metal coating that masks surface features [12–15]. The FS laser capsulotomies and manual CCC were only fixed in 2.5% glutaraldehyde solution for 24 hours and then imaged at low temperature (≤4°C) and high humidity (virtually 100%), thus greatly minimizing tissue dehydration and avoiding masking the microirregularities at the capsular edge.

The edges of manually torn capsules were smoother than the FS laser capsulotomy edges; no measurable differences in irregularity were found between the LenSx and the Victus specimens. The FS capsulotomies edges showed similar patterns, despite the intrinsic differences in laser settings and proprietary technology. Authors in [13–15] have shown that, using the LenSx laser, smoother cutting edges could be obtained by reducing the spot energy and by placing a soft contact lens between the cornea and the curved rigid interface; however, microdiscontinuities (e.g., cracks and tags) have been still shown when using low pulse energy.

The microscopic features and irregularity of the FS capsulotomy edges can be directly related both to photodisruption and to eye movements [12–15]. The photodisruptive mechanical and thermal effects contribute to the corrugating and stretching of the capsular edge [27–29], offering a mechanical basis for weakness in capsular integrity. These irregularities have been postulated to either limit the distension of the capsule or act as focal points for the concentration of stress that would increase the risk of capsular tear [12–15]. Auffarth et al. [30] have found, in porcine eyes, that FS laser capsulotomies resulted in a stronger anterior capsular opening than manual CCC, offering a hypothesis that tears may originate by increased stress at the capsular edges when pulling the capsulotomy leaf. On the other hand, biomechanical data from porcine specimens cannot be translated to the human lens capsule due to intrinsic differences in elasticity between species [18, 23–25, 31, 32]. Capsular tears in FS laser assisted cataract surgery have been mainly reported to occur with hydrodissection and during lens manipulations [12], suggesting that reduced capsular distensibility may represent an additional factor to increased risk of capsular tears during FS laser assisted cataract surgery. On the other hand, there is still no evidence supporting this hypothesis [17–19, 23–25, 33–36].

Eye movements during surgery (that are in the range between 20 and 100 *μ*m) have been considered to contribute to increased capsulotomy edges' irregularities, by creating multiple, random cavitations that could compromise the integrity of the capsular edge and represent a point for a tear to initiate with adequate force [12] during the capsulotomy pulling, hydrodissection, or nucleus manipulations. In this study we showed, for the first time, microdiscontinuities at the capsular edge (i.e., linear cracks; ≤3 *μ*m width). These features may originate by imprecise impact of the laser pulses with the lens capsule, likely due to eye movements during laser surgery, and may represent the real risk to generate tears in the case of increased capsular stress during FS laser assisted phacoemulsification. Further studies on the biomechanics of the human lens capsule, in relation to FS laser parameters and capsulotomy size and centration [37], are needed to understand the influence of FS photodisruption on capsular tears and how to create the capsulotomy edges with quality and morphology comparable to manually torn capsules [13, 14, 38]. Some irregularities in* ex vivo* studies may have arisen by increased IR absorption and scattering of donor corneal tissues, thus enhancing the differences between manually torn capsules and FS laser capsulotomy cutting edges characteristics.

In this study, we confirmed that the LEC boundary is closer to the manual CCC edge than FS capsulotomy edge. The results were in agreement with previous work [13–15]. Increased LEC death and inhibition of LEC proliferation may be beneficial for preventing PCO.

In conclusion, the FS laser capsulotomy edges show distinct irregularities, independent of the laser platform, that may be at risk of increased capsular tears during phacoemulsification. During the learning curve, the cataract surgeons should be conservative when pulling the capsule, during hydrodissection and nucleus manipulations. The implementation of robust eye tracking system in the FS laser platforms would greatly improve the smoothness of capsulotomy edges.

## Figures and Tables

**Table 1:** Irregularity analysis (mean ± SD) of the FS laser capsulotomy and manual CCC cutting edges.

	Sa (*µ*m)^*^	Sq (*µ*m)^*^	Ssk (no unit)	Sku (no unit)
LenSx capsulotomies (*n* = 5)	5.98 ± 0.56	1.18 ± 0.24	0.35 ± 0.24	−0.43 ± 0.63
Victus capsulotomies (*n* = 5)	6.49 ± 0.33	1.32 ± 0.16	0.63 ± 0.24	0.00 ± 0.42
Manual CCC (*n* = 5)	4.94 ± 0.56	0.57 ± 0.13	0.39 ± 0.14	0.44 ± 0.65

^*^Statistically significant differences between groups: *P* < 0.05.
